# Immunosuppressive Therapy of Biopsy-Proven, Virus-Negative, Autoimmune/Immune-Mediated Myocarditis—Focus on Azathioprine: A Review of Existing Evidence and Future Perspectives

**DOI:** 10.3390/biology12030356

**Published:** 2023-02-24

**Authors:** Justyna Grzechocińska, Agata Tymińska, Andrea Silvio Giordani, Julia Wysińska, Ewa Ostrowska, Anna Baritussio, Alida Linda Patrizia Caforio, Marcin Grabowski, Renzo Marcolongo, Krzysztof Ozierański

**Affiliations:** 1 First Department of Cardiology, Medical University of Warsaw, 1a Banacha St., 02-097 Warsaw, Poland; 2 Cardiology, Department of Cardiac Thoracic Vascular Sciences and Public Health, University of Padova, 35-100 Padova, Italy

**Keywords:** myocarditis, inflammatory cardiomyopathy, immunosuppressive therapy, endomyocardial biopsy, systemic immune-mediated diseases

## Abstract

**Simple Summary:**

Myocarditis is one of the leading causes of acute and chronic heart failure, adverse ventricular remodelling, and progression to dilated cardiomyopathy, life-threatening arrhythmias, and sudden cardiac death. According to recent guidelines, azathioprine, in association with steroids, is a cornerstone of first-line therapy regimens in biopsy-proven, autoimmune/immune-mediated, virus-negative myocarditis. Despite that the majority of published clinical studies seem to show an overall benefit of immunosuppressive therapy in the treatment of myocarditis/inflammatory cardiomyopathy, a targeted therapy has still not been standardized, and there is a need for further controlled, multicentric, clinical studies to provide further data on the efficacy and safety of IT in myocarditis. The aim of this review is to describe the pharmacological properties of azathioprine and to explore future perspectives for its usage in the cardioimmunology field.

**Abstract:**

The use of immunosuppressive therapy (IT) in biopsy-proven, autoimmune/immune-mediated (AI), virus-negative myocarditis has become the standard of care. In particular, according to recent guidelines, azathioprine (AZA), in association with steroids, is a cornerstone of first-line therapy regimens. IT may have a crucial impact on the natural history of AI myocarditis, preventing its progression to end-stage heart failure, cardiovascular death, or heart transplantation, provided that strict appropriateness and safety criteria are observed. In particular, AZA treatment for AI virus-negative myocarditis requires the consideration of some crucial aspects regarding its pharmacokinetics and pharmacodynamics, as well as a high index of suspicion to detect its overt and/or subclinical side effects. Importantly, besides a tight teamwork with a clinical immunologist/immuno-rheumatologist, before starting IT, it is also necessary to carry out a careful “safety check-list” in order to rule out possible contraindications to IT and minimize patient’s risk. The aim of this review is to describe the pharmacological properties of AZA, as well as to discuss practical aspects of its clinical use, in the light of existing evidence, with particular regard to the new field of cardioimmunology.

## 1. Introduction

Myocarditis is one of the leading causes of acute and chronic heart failure, adverse ventricular remodelling, and progression to dilated cardiomyopathy (DCM), life-threatening arrhythmias, and sudden cardiac death [1,2,3,4]. It is estimated that myocarditis affects thousands of patients annually worldwide, both of adult and paediatric age, with relevant medical and social consequences. Each year, more than 300,000 patients worldwide die by DCM resulting from chronic progression of myocarditis, with a 20–50% five-year mortality in the course of DCM [1,2,3,4]. Moreover, an increase in morbidity and mortality related to myocarditis has been recorded in recent decades, probably due to better recognition of the disease [5,6,7]. In fact, myocarditis may often be underdiagnosed, since in many cases the diagnostic gold standard, i.e., endomyocardial biopsy (EMB), is not performed [8].

Myocarditis can be caused by a wide range of agents, and the most relevant clinical distinction is between infectious and noninfectious forms [1,2,3,4]. Infectious agents may cause direct cardiomyocyte injury or trigger an autoreactive cellular and humoral immune response that leads to myocardial damage with inflammation [1]. Conversely, autoimmune/immune-mediated (AI) myocarditis may occur with exclusive cardiac involvement (i.e., organ-specific autoimmune disease) or in the context of systemic immune-mediated diseases (SIDs), such as eosinophilic granulomatosis with polyangiitis (EGPA), systemic lupus erythematosus (SLE), systemic sclerosis, and others [1,2,3,4,9]. Until recent years, noninfectious causes of myocarditis were less commonly reported than infectious ones, and a trend towards an increasing diagnosis of virus-negative myocarditis forms seems to be emerging [10].

Current international guidelines recommend the use of immunosuppressive therapy (IT) in virus-negative, AI myocarditis/inflammatory cardiomyopathy refractory to standard supportive optimal medical therapy, in patients without contraindications to IT [1,2,3,4]. So far, the drugs investigated in this setting are mainly steroids, azathioprine, cyclosporine, or mycophenolate mofetil, in different combinations [9,11,12,13,14,15,16].

In particular, azathioprine (AZA) has been extensively used due to its excellent profile of efficacy and safety as an immunosuppressive agent for the treatment of a variety of SIDs, such as SLE, rheumatoid arthritis, dermatomyositis, polymyositis, systemic sclerosis, and systemic vasculitis. Other possible indications for AZA, outside cardioimmunology, are inflammatory bowel diseases or prevention of organ transplant rejection [17]. The potential applications of AZA in the cardioimmunology field range from recurrent idiopathic pericarditis refractory to standard treatment [18,19], to virus-negative, AI myocarditis and inflammatory cardiomyopathy [10,14,15]. The introduction of the use of AZA in the treatment of AI inflammatory cardiomyopathy is relatively recent, but it is supported by robust evidence in form of RCTs with excellent results [12,20].

The scope of the present article is to describe the pharmacological properties of AZA, in the light of existing evidence on its use with biopsy-proven, virus-negative, AI myocarditis/inflammatory cardiomyopathy, and to explore future perspectives for its usage in the cardioimmunology field.

## 2. Pharmacological Properties of Azathioprine: A Focus on Clinical Implications

**Figure 1:**
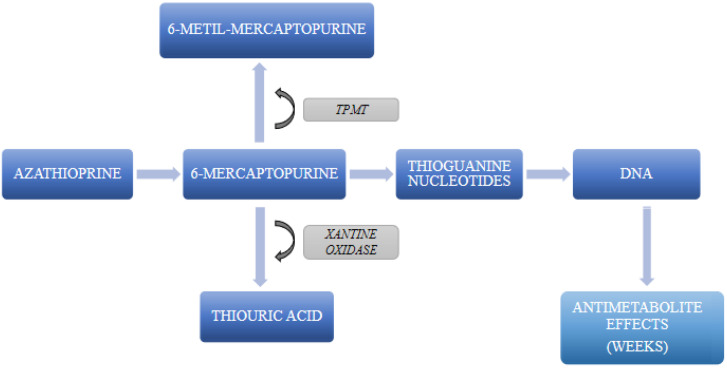
Diagram summarizing the crucial steps on AZA metabolism. TPMT: thiopurine methyltransferase.

AZA is an immunosuppressive agent orally administered as an inactive prodrug [17,21]. Once converted in the liver and kidneys to 6-mercaptopurine, its active form, it acts as an antimetabolite of purine bases. In particular, it interferes with DNA synthesis by incorporating purine thioanalogues into the DNA chain [17]. As a result, it inhibits the biosynthesis of nucleic acids and prevents the proliferation of immunocompetent cells. Following oral administration, AZA absorption varies between 27 and 80%, while its bioavailability decreases by about 26% if ingested with food. Moreover, AZA is degraded by xanthine oxidase in milk [17]. Therefore, this drug should be ideally administered at least one hour before or three hours after a meal or milk consumption. AZA binds to plasma proteins in a proportion of about 30% and is metabolized in the liver and kidneys, where it is rapidly broken down to 6-mercaptopurine and methylnitroimidazole [17]. 6-mercaptopurine readily enters the cells where it is transformed into purine thioanalogs [17]. It is finally converted into thiouric acid, an inactive metabolite, and, to a lesser extent, into 1-methyl-4-nitro-5-thioimidazole, which are excreted in the urine [17] (Figure 1). There are no sufficient data on the clearance and biological half-time of AZA. The half-life of 6-mercaptopurine is about 0.9 h; approximately 12% of AZA is excreted, unchanged, in the faeces, while a further 20–50% is eliminated, unchanged or as metabolites, with urine [17,22]. Notably, it may be only partially removed by dialysis.

Measurement of concentrations of AZA metabolites may be used to monitor compliance [23]. Although there were conflicting results as to whether there is a direct association between AZA metabolite concentrations and the probability of disease remission, it is recommended to monitor the AZA metabolite concentration (6-TGN and 6-MMP) when treated with AZA [24,25]. AZA metabolites accumulate in lymphocytes, block the expression of cytokines, and, finally, inhibit the inflammatory response induced by T cells [26].

The metabolism of 6-mercaptopurine is mediated by several enzymes, such as thiopurine methyltransferase (TPMT), xanthine oxidase, inosine monophosphate dehydrogenase, hypoxanthine-guanine phosphoribosyl transferase, and aldehyde oxidase [17,21]. Polymorphisms of genes encoding the different enzymes involved in the metabolism of AZA and in particular, TPMT may imply an increased risk of adverse effects during treatment [27], especially agranulocytosis, which is a rare but potentially lethal side effect of the drugs that justifies the screening for TPMT before starting AZA treatment [28,29]. TPMT deficiency is an autosomal codominant trait [30] and is diagnosed with genetic testing; of note, in patients who have received a blood transfusion in the previous three months, TPMT testing can be unreliable because of TPMT activity in the transfused blood cells [31]. Several reports exist on thiopurine-induced haematological toxicity, especially myelosuppression, in patients with inherited low TPMT activity treated with standard doses of AZA [32].

AZA immunosuppressive effect usually becomes gradually apparent within several weeks of treatment; this is of particular relevance in clinical practice because other agents may be administered in combination, usually high-dose steroids, to produce a rapid immunosuppressive action. Moreover, since AZA effects on the left ventricular function may take time to become evident, patients may need temporary measures, such as a wearable cardiac defibrillator (WCD), in the meantime [33]. Finally, in case of specific histotypes of myocarditis, such as giant-cell myocarditis (GCM), which was defined as “the most fatal of autoimmune diseases” [34], AZA therapy alone may not be sufficient, especially in the acute phase; therefore, AZA is always integrated in combination IT regimens for GCM [35].

## 3. The Use of Azathioprine in Clinical Practice: A Pragmatic Approach

In clinical practice, AZA is usually administered as a steroid-sparing agent to maintain remission following induction therapy in AI diseases and to prevent graft rejection [17]. AZA, especially in combination with corticosteroids, is approved for the treatment of:

- multiorgan involvement in SIDs, such as SLE, rheumatoid arthritis, dermatomyositis, polymyositis, periarteritis nodosa, pemphigus vulgaris, pyoderma gangrenosum, autoimmune haemolytic anaemia, chronic refractory thrombocytopenic purpura, and autoimmune chronic hepatitis [36];
- moderate-to-severe chronic inflammatory bowel diseases (IBDs), such as Crohn’s disease or ulcerative colitis [37];
- prevention of graft rejection after kidney, heart, or liver transplantation [38].

In most cases, a standard therapeutic dose usually ranges from 1 to 2 mg/kg per day, though organ transplantation may require a higher dose [17]. The dose should be adjusted within this range depending on the patient’s individual clinical response, which becomes evident after several weeks of treatment and according to haematological and hepatic tolerance. Based on the clinical experience developed in patients with SIDs, treatment with AZA is usually prolonged over 6 months, for at least 12 months or longer. Accordingly, the same treatment duration can be adopted also for patients with biopsy-proven, virus-negative, AI myocarditis/inflammatory cardiomyopathy, even if evidence so far has focused on shorter therapy intervals [12,20]. Recent guidelines, in fact, recommend IT for at least 6–12 months in selected biopsy-proven AI myocarditis patients with unremitting HF [2]. After ruling out TPMT mutations, it is advisable to introduce AZA treatment gradually, starting with 1 mg/kg per day to test its haematological, hepatic, and pancreatic tolerance, until reaching the target dose in about two weeks. The dose should be then titrated on an individualized basis, depending on clinical response, tolerance, and additional criteria (patients >65 years of age, comorbidities, fragility, and/or body weight ≤60 kg; haematological response, particularly leukopenia; and pancreatic, hepatic, and gastrointestinal tolerance, etc.). Possible adverse effects of AZA are bone-marrow depression (leukopenia, anaemia, and thrombocytopenia), nausea, and viral and opportunistic infections; hypersensitivity reactions; pancreatitis, hepatitis, and cholestasis; and malignancies [17]. The reported adverse events of AZA are more common among patients receiving high doses of the drug (e.g., after organ transplantation) and/or with reduced tolerance due to a TPMT mutation [17]. Consequently, patients with TPMT mutations should be switched to an alternative drug.

AZA, when correctly used, is usually a safe drug. Nevertheless, focusing on its employment in the cardioimmunology field, the rate of side effects of any grade may be sizeable (up to 20%, according to literature [39]); therefore, periodic clinical and laboratory reassessment of treated patients is advisable, preferably with supervision by a clinical immunologist/rheumatologist with expertise in the field. In fact, the timing of occurrence of AZA side effects (especially subclinical liver toxicity detected by lab tests) is greatly variable and unpredictable, with onset even up to several months from drug initiation. From a practical point of view, frequent monitoring of complete blood-cell count and liver-function test is recommended during the first 4 to 8 weeks, and then every three months for the remaining treatment period after reaching the maintenance dose. In special populations (chronic kidney disease, elderly patients, and high AZA dosages), lab tests should be performed more frequently [40]. Finally, some authors suggest monitoring the level of AZA metabolites to avoid specific complications [41].

## 4. Existing Evidence on Azathioprine Effectiveness and Safety in Myocarditis and Inflammatory Cardiomyopathy

Current guidelines [2] suggest IT for the treatment of biopsy-proven, virus-negative, AI myocarditis/inflammatory cardiomyopathy (e.g., lymphocytic, eosinophilic, sarcoid, and giant-cell myocarditis) with severe myocardial function impairment and/or life-threatening arrhythmias [42]. A list of the results of clinical trials testing IT on AI myocarditis/inflammatory cardiomyopathy is reported in Table 1 [12,13,14,15,43,44].

The first randomized clinical trial investigating the role of AZA in the IT of myocarditis was the Myocarditis Treatment Trial in 1995. Following a 6-month treatment with cyclosporine or AZA, in combination with prednisone, when compared with placebo, this study showed no benefits. A main limitation of this study was that it did not distinguish viral from nonviral forms of myocarditis; in fact, EMBs were only analysed according to the histological Dallas criteria, without undergoing a viral genome search. In a retrospective analysis [47], the virological and immunological characterization of lymphocytic myocarditis patients treated with IT revealed a 90% responsiveness rate in virus-negative cases, whilst a viral genome was detectable in the myocardium of 85% of nonresponders. Moreover, this trial was underpowered for detecting survival differences [46].

Conversely, from the year 2000 and onwards, some single-centre studies, based on small patient populations of biopsy-proven myocarditis patients, reported beneficial effects of AZA in combination with prednisone on left ventricular (LV) ejection fraction (EF), with excellent results (improvement in up to 90% of patients), and a very high safety profile; in fact, no significant adverse events were reported [12,13].

In 2001, Wojnicz et al. randomized 84 patients with DCM and increased human leukocyte antigen (HLA) expression on EMB for a 3-month treatment with AZA and prednisone versus placebo [13]; the presence of a viral genome was not assessed. After 3 months, 71.8% of patients in the IT group versus 20.9% of patients in the placebo group met the criteria of improvement, i.e., increase in LVEF, reduction in LV diastolic dimension and volume, and reduction in New York Heart Association (NYHA) functional class (*p* < 0.001).

In 2009, Frustaci et al. published the first randomized, double-blind, placebo-controlled trial including patients with biopsy-proven, virus-negative myocarditis and chronic (>6 month) heart failure unresponsive to conventional therapy [12]. In the IT group, 43 patients received prednisone (1 mg/kg body weight per day for 4 weeks, followed by 0.33 mg/kg body weight for 5 months, i.e., 6 months in total) and AZA (2 mg/kg body weight for 6 months); 42 patients were randomized to the placebo group. The primary outcome was a 6-month improvement in LVEF assessed by echocardiography. Secondary objectives were the improvement of NYHA class and survival from cardiac death or heart transplantation. This study demonstrated a significant improvement of LVEF and a significant decrease in LV dimensions and volume compared with baseline in 88% of patients in the treatment group. Even patients with a severely reduced systolic function at baseline (LVEF < 20%) and severe LV adverse remodelling (LV end-diastolic diameter up to 90 mm) showed a substantial benefit from IT. A remaining minor, yet relevant, quote of 12% of patients demonstrated a stable clinical picture with no improvement of cardiac function parameters. Frustaci et al. attributed the lack of response to IT of this subset of patients to the possible lack of exclusion of presence of viruses that were not screened at baseline and to mechanisms of myocardial damage not targeted by IT. Another remarkable result is that 49% of the patients receiving IT improved by at least one NYHA class at 6 months. In contrast, none of the patients in the placebo group showed improvement of LVEF nor NYHA class. Nevertheless, at one month from baseline, 38% of patients in the placebo group showed a mild improvement of LVEF, which lasted up to 3 months, but then it declined to baseline or even lower values.

In the trials by Wojnicz and Frustaci, the duration of IT with prednisone and AZA ranged from 3 to 6 months, respectively [12,13]. As previously mentioned, both studies showed a positive effect of IT on both echocardiographic parameters and physical recovery. Recently, Chimenti et al. published a 20-year follow up of the TIMIC trial that confirmed the lasting benefit of IT, both in terms of LV function and of survival from death and heart transplant [20]. However, since the clinical effect of AZA takes 1 to 3 months to become completely evident, it is conceivable that a further improvement could be expected, prolonging the treatment to 12 months or more [48,49].

Another study by Maisch et al. (the European Study on the Epidemiology and Treatment of Cardiac Inflammatory Disease), a double-blind, randomized, placebo-controlled trial IT with prednisolone and AZA was effective in patients with AI, virus-negative, inflammatory dilated cardiomyopathy with an LVEF <45% [50]. Following 6 months of treatment, a significant improvement was observed in both LVEF and major adverse cardiac events, which was still lasting after 1 year of follow-up. Remarkably, the control group also showed some spontaneous resolution.

## 5. Safety Check-List before Starting IT in Biopsy-Proven AI Myocarditis/Inflammatory Cardiomyopathy

Apart from a histological confirmation of the absence of a viral genome on EMB, another fundamental prerequisite for IT in myocarditis is the verification of a lack of contraindications to IT [1]. In order to help clinicians to identify possible IT contraindications, a detailed “safety checklist” has been published [51] (Table 2). This set of investigations is intended to be performed before starting IT in all patients, aiming at the identification of absolute contraindications and individual risks related to IT [52]. Patients should be screened for common latent infections and hidden malignancies, taking into account the individual patient’s characteristics, such as ethnicity, sex, and age (for example, reproductive age, or advanced age with increased frailty profile). As previously outlined, of particular importance is the search for the mutation of TPMT before starting AZA, to prevent the appearance of secondary leukopenia/agranulocytosis. In addition, IT of AI myocarditis requires a multidisciplinary approach (i.e., cardiologists, cardiac pathologists, immunologists, rheumatologists, and other professionals) and an active involvement of the patients and their caregivers.

## 6. AZA Therapy in Cardioimmunology: Existing Evidence and Future Perspectives

Based on the pathophysiology of inflammatory heart diseases, it is assumed that a disease-specific treatment should include IT to put under control the adverse immune responses taking place in the myocardial tissue [1,10]. Most available clinical studies seem to show an overall benefit from IT in the treatment of AI, biopsy-proven, virus-negative myocarditis, but the targeted therapy has still not been specified. Published studies have mostly presented inhomogeneous patient populations (small, underpowered groups) [53] at a different disease stage (acute vs. chronic myocarditis) [14,50], often with an undefined type of cellular infiltration (e.g., lymphocytic) and/or of unknown viral presence in heart biopsy [13,53].

The nature of the above-mentioned data makes it difficult to perform a qualitative meta-analysis; therefore, those analyses that were conducted showed conflicting data [54,55]. Most of the beneficial results of IT relate to combined treatments with prednisone and AZA on top of optimal guideline-based medical therapy for HF. These results seem to be derived from the complementary pharmacokinetic properties of these medications. Prednisone usually is started at high doses to induce the rapid suppression of myocardial inflammation and the control of pathological immune response, while the immunosuppressive effect of AZA becomes gradually apparent only after several weeks of treatment, playing a crucial role in maintaining the effects of induction therapy and preventing relapses, while tapering down steroid dosage [49]. However, some crucial questions are still waiting for answers about the most appropriate moment to start IT in AI, biopsy-proven, virus-negative myocarditis/inflammatory cardiomyopathy and when to stop it. Despite that the majority of published clinical studies seem to show an overall benefit of IT in the treatment of AI, biopsy-proven, virus-negative myocarditis/inflammatory cardiomyopathy, a targeted therapy has still not been standardized, and there is a need for further controlled, preferably multicentric, clinical studies to provide further data on the efficacy and safety of IT in myocarditis [3,4,56]. Currently, a multicentric, double-blind, randomized trial (IMPROVE-MC) on a combined 12-month therapy of AZA with prednisone is ongoing in Poland [57]. The study also aims to assess the safety and long-term effects of the treatment after completing a full course of the therapy.

Further studies are needed to improve the selection of patients (i.e., based on disease aetiology, disease activity, and presence of anti-heart autoantibodies and specific inflammatory cells/cytokines) who would mostly benefit from the IT treatment [58,59]. What is more, in the future, a therapeutic-drug monitoring of thiopurine metabolites could improve clinical outcomes through dose optimization and toxicity monitoring [60].

## 7. Conclusions

Existing evidence shows that IT is of paramount importance in the management of biopsy-proven, virus-negative, AI myocarditis/inflammatory cardiomyopathy in patients that do not respond to conventional cardiovascular medical treatments. Azathioprine is one of the most studied drugs in this setting. If correctly managed, AZA is a safe and effective tool to modify myocarditis’s natural history, preventing its progression to DCM, end-stage HF, death, or HTx.

## Figures and Tables

**Table 1:** Summary of the main characteristics of trials exploring the use of AZA, in association with prednisone, in biopsy-proven, virus-negative myocarditis/inflammatory cardiomyopathy.

Study	Type of Study and Number of Patients	Histological Type and PCR for Virus	Indications for Therapy	AZA Dosage and Duration of Treatment	Control Group	Additional Treatment	Major Outcomes and Mortality
Salvi 1989 [45]	Observational, uncontrolled, and longitudinal; 20 pts.	Unknown and unknown.	Myocarditis.	75 mg/m^2^/day for at least 6 months	-	PRED: 50 mg/m^2^/day for 2 weeks, then tapered until withdrawal of the drug after at least 6 months of treatment; + digoxin, diuretics, vasodilators, and amiodarone were continued (if started earlier), metoprolol when tachycardia or hypertension.	Histologic status improved in all pts, complete disappearance of signs of active disease in 15 pts. An overall improvement of LVEF (from 0.37 +/− 0.14 to 0.46 +/− 0.17). Direct relationship between the histological changes and the changes in LVEF in some pts. 2 deaths during observation (both pts with low and deteriorating LVEF from 0.26 to 0.16), 1 death after the end of treatment due to cerebral haemorrhage during anticoagulant treatment.
Frustaci 2009 [12]	Randomized, double-blind, and placebo-controlled; 85 pts (43 vs. 42 placebo).	Lymphocytic andvirus-negative.	Virus-negative myocarditis and chronic (>6 months) HF unresponsive to conventional therapy.	2 mg/kg/day for 6 months	Placebo + optimal conventional treatment for HF with ACEI, β-blockers, and diuretics.	PRED: 1 mg/kg/day for 4 weeks, followed by 0.33 mg/kg/day for 5 months; + optimal conventional treatment for HF with ACEI, β-blockers, and diuretics.	IT group: a significant improvement of LVEF and a significant decrease in LV dimensions and volumes compared with baseline; recovery of LV function in 88% of pts with no case of death or HTx during treatment; in the following 6 months, improvement in patients with extreme LV dilatation and dysfunction accompanied at histological examination by the disappearance of inflammatory infiltrates with progression of the disease from an active towards a healed myocarditis. Placebo group: initial improvement in some pts, further impairment of cardiac function in 83% of pts; 2 pts received transplants and 2 pts died in the 6 months after the end of trial.
Escher 2016 [14]	Observational, retrospective, uncontrolled, and longitudinal; 114 pts.	Lymphocytic andvirus-negative.	EMB-proven, virus-negative chronic myocarditis or ICM. All pts had symptoms of HF of unknown cause for at least 6 months, despite more than 2 months of stable clinical status and stable optimal conventional HF therapy (including ACEI, β-blockers, and diuretic).	2 mg/kg/day for 6 months	-	PRED: 1 mg/kg/day for 4 weeks, followed by 0.33 mg/kg/day for 5 months; + stable optimal conventional therapy for chronic HF (no relevant changes in medication for chronic HF were allowed that either would have been expected to be given to further improve the patient’s clinical symptoms at the time of enrolment, or that would have become necessary due to a marked deterioration of chronic HF within 8 weeks before enrolment).	Significant improvement of LVEF compared to baseline; these effects lasted for the extended long-term follow-up period. At follow-up EMB, a significant decrease in CD3^+^ lymphocytes/m^2^ as well as an abundance of the HLA-1 could be observed; in all of the patients, perforin^+^ cells and CD2^+^ cells decreased significantly in a similar manner, in comparison to baseline EMB.
Wojnicz 2001 [13]	Randomized and placebo-controlled; 84 pts (41 vs. 43 placebo); final assessment: 58 (28 vs. 30 placebo).	Unknown and unknown.	Chronic myocarditis.	1 mg/kg/day for 100 days	Placebo.	PRED: 1 mg/kg/day, for 12 days, then tapered every 5 days by 5 mg/day until reaching the maintenance dose of 0.2 mg/kg/day for a total of 90 days; + furosemide, spironolactone, captopril, metoprolol tartrate, nitrates, and amiodarone hydrochloride.	Significant LVEF increase, improvement of LV volume, LV diastolic dimension, and NYHA class in the IT group compared with the placebo group after 3 months of follow up. 5 out of 84 pts (5.9%) died, 6 pts (7.1%) underwent HTx, and 5 (5.9%) were readmitted to hospital during the 2-year period.
Mason 1995 [46]	Nonrandomized and controlled; 111 pts.	Unknown and not assessed.	Myocarditis and LVEF <45%.	2 mg/kg/day for 24 weeks	1st group: placebo + conventional therapy for HF; 2nd group: CsA + PRED + conventional therapy for HF; CsA: 5 mg/kg twice daily, adjusted to achieve a blood level of 200–300 ng/mL at the end of week 1, then tapered to achieve a blood level of 100–200 ng/mL during weeks 2–4. From the end of week 4 to the end of week 24, the blood level was maintained at 60–150 ng/mL; PRED: 1.25 mg/kg/day for 1 week, then rapidly tapered to 0.15 mg/kg/day by the end of week 3 and maintained through week 23, then halved for a week and discontinued at the end of week 24.	PRED: 1.25 mg/kg/day for 1 week, then decreased by ~0.08 mg/kg/week until the dose was 0.33 mg/kg/day at the end of week 12. This reduced dose was maintained through the end of week 20, after which it was reduced by 0.08 mg/kg/week until the end of week 24, when the drug was discontinued; + conventional therapy for HF.	Ventricular function improved regardless of treatment (mean LVEF 0.25 ± 0.01 at baseline vs. 0.34 ± 0.02 at 28 weeks). No beneficial effect of IT on the primary endpoint (a change in the LVEF at 28 weeks) was observed. IT had a statistically significant (though clinically mild) negative influence on the LV internal diameter at end diastole. The two groups did not differ significantly in survival. The mortality rate was 20% at 1 year and 56% at 4.3 years for the whole group.
Merken 2018 [15]	Retrospective and nonrandomized, 1:1 propensity score-matching; 209 pts (110 vs. 99 placebo).	Unknown and virus-negative.	Virus-negative, nonfulminant ICM.	2 mg/kg/day for at least 6 months (median 6.3 months; mean 7.2 months).	Optimal conventional HF medication, including ACEI and β-blockers.	PRED: 1 mg/kg/day with a progressive step-down regimen after 1 month. CsA: 150 mg daily added in 11 cases for at least 6 months based on the immune profile in blood and EMB, such as highly elevated soluble interleukin 2 or neopterin.	After a median follow-up of 31 (15–47) months, IT resulted in an improved long-term outcome (e.g., HTx–free survival) as compared with standard HF therapy alone, and a significantly larger increase in LVEF after a mean of 12 months of follow-up, as compared with pts receiving standard HF treatment. 3 pts died: 1 within 1 month (no IT), 1 within 4 months (IT regimen), and 1 within 11 months (IT regimen, due to pulmonary cancer).
Jones 1991 [43]	Observational, uncontrolled, and longitudinal; 20 pts.	Unknown and unknown.	9 patients with EMB-proven myocarditis and 11 patients with borderline myocarditis.	1.5 mg/kg/day for 6–8 weeks (AZA discontinued 2 weeks after discontinuation of PRED).	PRED: 1 mg/kg/day tapered over the following 6 to 8 weeks.	-	Significant LV function improvement in the group with borderline myocarditis and no significant changes in the myocarditis group. No deaths or irreversible complications due to IT.
Poloczkova 2022 [44]	Prospective, randomized, and multicentre; 20 pts (9 vs. 11 with HF treatment only). The final analysis compared a group of patients treated with combined IT (regardless of the scheme) in addition to the conventional HF therapy and that of patients on conventional HF therapy only.	Unknown and virus-negative.	EMB-proven ICM and negative viral genome findings (except PVB19 low viral load presence < 500 copies/μg genomic DNA).	1st arm: 1 mg/kg/day for 100 days2nd arm: 2 mg/kg/day for 6 months.	Conventional HF treatment: ACEI or ARB, β-blockers, and spironolactone.	1st arm: PRED + conventional therapy for HFPRED (90 days): 1 mg/kg/day for 12 days, then tapered every 5 days by 5 mg/day down to 0.2 mg/kg/day 2nd arm: PRED + conventional therapy for HF PRED (6 months): 1 mg/kg/day for 4 weeks, followed by a dose of 0.33 mg/kg/day for the remaining 5 months.	No positive effect of combined IT on the LV function over 12 months. The baseline values of LVEF in the group of IT (LVEF 22.3 ± 4.7%) were similar to those in the group treated with conventional HF therapy (LVEF 21.7 ± 4.7%; *p* = 0.757). After 12 months there was no statistically significant difference in LVEF between the two studied groups (LVEF 33.7 ± 9.5% for the IT group and 41.3 ± 13.0% for the conventional therapy group; *p* = 0.175). 1 death from a non-CV cause in the IT-treated group (generalized cancer of unknown origin).
Chimenti 2022 [20]	Retrospective and nonrandomized, with 1:2 propensity score-matching; 85 (Group A—TIMIC trial pts [12]) vs. 170 (Group B—1:2 propensity score-matched control cohort of pts untreated with the TIMIC protocol).	Unknown and virus-negative.	EMB-proven diagnosis of virus-negative chronic ICM.	2 mg/kg/day for 6 months	optimal conventional HF therapy.	PRED 1 mg/kg/day for 4 weeks followed by 0.33 mg/kg/day for 5 months; +optimal conventional HF therapy.	At long-term follow-up, the risk of CV death (HR 6.77; 95% CI 2.36–19.45) and HTx (HR 7.92; 95% CI 1.80–34.88) was significantly higher in Group B. Group A showed a persistent improvement in the LVEF compared with Group B (HR 7.24; 95% CI 3.05–17.18). A higher number of Group B pts underwent ICD implantation. The incidence of recurrent myocarditis was similar between groups, and patients with evidence of a recurrent cardiac inflammatory process promptly responded to a TIMIC protocol application. CV deaths: 4 in Group A; 48 in Group B.

ACEI—angiotensin-converting enzyme inhibitor; ARB—angiotensin receptor blocker; AZA—azathioprine; CD—cluster of differentiation; CI—confidence interval; CsA—cyclosporine A; CV—cardiovascular; EMB—endomyocardial biopsy; HF—heart failure; HLA—human leukocyte antigen; HR—hazard ratio; HTx—heart transplant; ICD—implantable cardioverter–defibrillator; ICM—inflammatory cardiomyopathy; IT—immunosuppressive therapy; LV—left ventricle; LVEF—left ventricular ejection fraction; NYHA—New York Heart Association; PCR—polymerase chain reaction; PRED—prednisone; pts—patients; PVB19—parvovirus B19, TIMIC—tailored Immunosuppression in virus-negative inflammatory cardiomyopathy.

**Table 2:** Proposed “safety check-list” prior to initiation of IT. Modified from R. Marcolongo et al. [51].

**Laboratory Testing**
Complete blood cell count
Erythrocyte sedimentation rate, C reactive protein
Renal and liver function
Fasting glucose levels
Serum immunoglobulin levels
NT-pro-BNP/BNP, Troponin I/T
Serum pancreatic amylase (if azathioprine is planned)
Serological screening for latent infections (HBV, HCV, HIV, CMV, EBV, tuberculosis (QuantiFERON), Borreliosis, etc.)
Thiopurine methyltransferase (TMPT) mutation (if azathioprine is planned)
Pregnancy test (if appropriate)
Serum Prostatic Specific Antigen (PSA) (if appropriate)
**Imaging testing**
Chest X-ray
Abdominal ultrasound scan (if appropriate)
Gynaecological inspection/cervical smear examination
Screening mammography (if appropriate)

BNP—B-type natriuretic peptide; CMV—cytomegalovirus; EBV—Epstein–Barr virus; HBV—hepatitis B virus; HCV—hepatitis C virus; HIV—human immunodeficiency virus; NT-pro-BNP—N-terminal pro-B-type natriuretic peptide.

## Data Availability

Publicly available datasets were analysed in this study.
